# From Intermolecular Interactions to Texture in Polycrystalline Surfaces of 1,ω-Alkanediols (ω = 10–13)

**DOI:** 10.3390/molecules22060956

**Published:** 2017-06-08

**Authors:** Gilgamesh Luis-Raya, Màrius Ramírez-Cardona, Gabriel Luna-Bárcenas, Martín A. Hernández-Landaverde, Adair Jiménez-Nieto, Jose Luis García-Rivas, Beatriz Liliana España-Sánchez, Isaac C. Sanchez

**Affiliations:** 1 Área Académica de Ciencias de la Tierra y Materiales, Universidad Autónoma del Estado de Hidalgo, Ciudad del Conocimiento, Col. Carboneras, Mineral de la Reforma, 42184 Hidalgo, Mexico; gilura6969@hotmail.com or gilgamesh@upp.edu.mx; 2 Universidad Politécnica de Pachuca, Carretera Pachuca-Cd. Sahagún km 20 Ex-Hacienda de Santa Bárbara, Zempoala, 43830 Hidalgo, Mexico; 3 Centro de Investigación y Estudios Avanzados, Unidad Querétaro, Querétaro, 76200 Querétaro, Mexico; martinhernandez@cinvestav.mx (M.A.H.-L.); adairjn@cinvestav.mx (A.J.-N.), lespana@cinvestav.mx (B.L.E.-S.); 4 División de Estudios de Posgrado e Investigación, Instituto Tecnológico de Toluca, Av. Tecnológico s/n Colonia Agrícola Bella Vista, 52149 Metepec, Mexico; lgrivas230@hotmail.com; 5 Department of Chemical Engineering, The University of Texas at Austin, Austin, 78712-1589 TX, USA; sanchez@che.utexas.edu

**Keywords:** Glancing Incidence X-ray diffraction, multiaxial preferred orientation, Rietveld refinement, C-H···O and O-H···O hydrogen bonds, alkanediol forms and polytypes

## Abstract

Differences on *herringbone* molecular arrangement in two forms of long-chain 1,ω-alkanediols (C_*n*_H_2*n*+2_O_2_ with *n* = 10, 11, 12, 13) are explained from the analysis of O-H···O hydrogen-bond sequences in infinite chains and the role of a C-H···O intramolecular hydrogen-bond in stabilization of a *gauche* defect, as well as the inter-grooving effectiveness on molecular packing. GIXD (Glancing Incidence X-ray Diffraction) experiments were conducted on polycrystalline monophasic samples. Diffracted intensities were treated with the multi-axial March-Dollase method to correlate energetic and geometrical features of molecular interactions with the crystalline morphology and textural pattern of samples. The monoclinic (P*2*_1_/*c*, *Z* = 2) crystals of the even-numbered members (*n* = 10, 12; DEDOL and DODOL, respectively) are diametrical prisms with combined form {104}/{-104}/{001} and present a two-fold platelet-like preferred orientation, whereas orthorhombic (P*2*_1_*2*_1_*2*_1_, *Z* = 4) odd-numbered members (*n* = 11, 13; UNDOL and TRDOL, respectively) present a dominant needle-like orientation on direction [101] (fiber texture). We show that crystalline structures of medium complexity and their microstructures can be determined from rapid GIXD experiments from standard radiation, combined with molecular replacement procedure using crystal structures of compounds with higher chain lengths as reference data.

## 1. Introduction

The compounds of the 1,ω-alkanediols (C_*n*_H_2*n*+2_O_2_) series with *n* ≥ 9 crystallize at low temperatures into two ordered forms according to the parity of carbon atom numbers in the chain [1]. 1,ω-alkanediols with an even number of carbons crystallize into a structure with layers of the type *herringbone* (monoclinic with the space group P*2*_1_/*c*, *Z* = 2), where interlayer hydrogen bonds form *zigzag* chains. Odd-numbered compounds (orthorhombic with space group of P*2*_1_*2*_1_*2*_1_, *Z* = 4) also present a molecular packing in layers but, so far, they have been described with molecules oriented parallel to each other [2,3,4,5,6,7,8,9,10,11,12,13]. In this case, the pattern of hydrogen bond is formed by alternation of inter and intralayer O-H···O.

The presence of the terminal (-OH) polar groups results in molecular aggregation and surfactant properties, which lead to the formation of layers or films in the air-water interface [14]. These aggregates are presented in the form of amphiphilic monolayers (2-D), or nano-crystalline multilayer aggregates (3-D) [15]. Its important interactivity with water does not preclude its solubility in mixed micelles [16]. Moreover, alkanediols are an important component in epicuticular wax crystals in plant surfaces e.g., [17], with a significant hydrophobic function. In this sense, these complex systems (i.e., aggregates and biological structures) offer an invaluable opportunity to conduct and prove in situ experiments by means of surficial characterization techniques so as to elucidate morphology and structure of molecular crystals, such as the compounds object of this study. To perform a molecular array study in this kind of molecular nanostructure, it is customary to use crystallographic characterization techniques at the surface level, for example, Grazing Incidence X-ray Diffraction (GIXD, or Glancing instead of Grazing for angles up to a few degrees [18]). In comparison with Bragg–Brentano geometry, GIXD keeps a fixed incident angle (asymmetric configuration) and provides an easier way to detect preferred orientations when several experiments at different incident angles are performed. This feature may also be useful for the structural study of organic compounds with a remarkably molecular anisotropy (e.g., long or medium-chain aliphatic compounds: n-alkanes, n-alcohols, fatty acids or derived) and with crystal packing arrangements in layers because, chiefly in flat specimens, their polycrystalline samples are usually oriented along planes with long interplanar spacing. During GIXD experiments using standard laboratory radiation and combining a relatively low incident angle and a parallel beam, it is assured higher intensities than the conventional geometry of Bragg–Brentano.

The goal of this work is to prove the viability of using data obtained from rapid experiments with GIXD to determine the bulk (3D) crystal layered-structure of four 1,ω-alkanediols (ω = 10, 11, 12, 13). The procedure of structural determination consists of the following stages: (i) indexation and space-group determination; (ii) whole-pattern-fitting; (iii) isomorphic molecular replacement; (iv) lattice energy minimization and (v) rigid-body Rietveld refinement. To validate results, the structures obtained are compared with those previously reported by single-crystal X-ray diffraction [2,3,4,5,6,7,8,9,10,11,12]. Also, the value of lattice energies is compared with the corresponding sublimation energy. In the Rietveld refinement, a multiaxial March-Dollase preferred orientation study [19] is implemented to improve the “goodness-of-fit”. Taking advantage of this latter analysis, important textural differences can be detected between powdered samples of both polymorphs of the series.

Section 2 (Results and Discussion) of this article will present the polymorphic modifications within the series of 1,ω-alkanediols from comparisons at intermolecular and microstructural scales, based on results obtained by different characterization methods. Synthesis and preparation of materials, techniques and their experimental conditions are summarized in Section 3. Section 4 is a detailed description of the methods used in the crystal structure determination, from the indexation of GIXD pattern to the structural refinement. Finally, Section 5 is the concluding part where the more relevant challenges among results and methods are assembled.

## 2. Results and Discussion

### 2.1. From Hydrogen Bonds to Herringbone Angle

**Figure 1:**
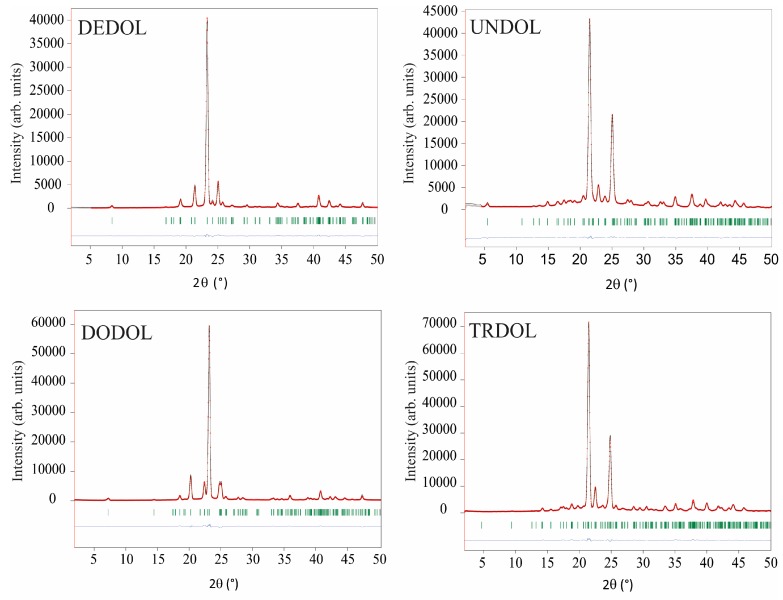
Rietveld fits between both experimental (red dots) and calculated (black lines) glancing incidence X-ray diffraction (GIXD) patterns. Blue line: differences curves. Green bars: Bragg positions.

Refinements of the four 1,ω-alkanediols crystal structures are illustrated by Rietveld plots (see Section 4, entitled “Structure Determination”, for a more detailed description of refined parameters) in Figure 1. These results from GIXD patterns show slight differences with those obtained earlier by single crystal X-ray diffraction [2,3,4,5]. That is to say, values of interatomic distances and angles fall, in general, within the range established on standardized tables [20]. Some covalent distances C-H of the C-H···O interaction result in notable deviations from normalized values, because of the lattice energy minimization process (i.e., without restrictions in bond lengths) and anomalous bond lengths in the starting structural models; a situation that it is not easy to avoid due to the characteristic limitation and precision of the powder X-ray diffraction data obtained by rapid experiments using laboratory radiation. On the other hand, a good accuracy in the determination of the position of H atoms in hydrogen bonds is obtained if both DFT calculations and 1H NMR chemical shifts are used as complementary methods to X-ray or even for neutron diffraction data [21,22,23].

The terminal torsion angle, HO-C-C-C, is close to 180° for DEDOL (176.11°) and DODOL (179.10°). This isomer is named as TTt, in reference to the conformations on the three torsion angles involved along the molecular axis [24] in its terminal segment, H-O-C-C-C-C. UNDOL and TRDOL molecular structures present in one of the final segments a torsion angle close to 180° (176.20° and 179.80°, respectively), whereas the other is 64.01° for UNDOL and 66.64° for TRDOL.

**Figure 2:**
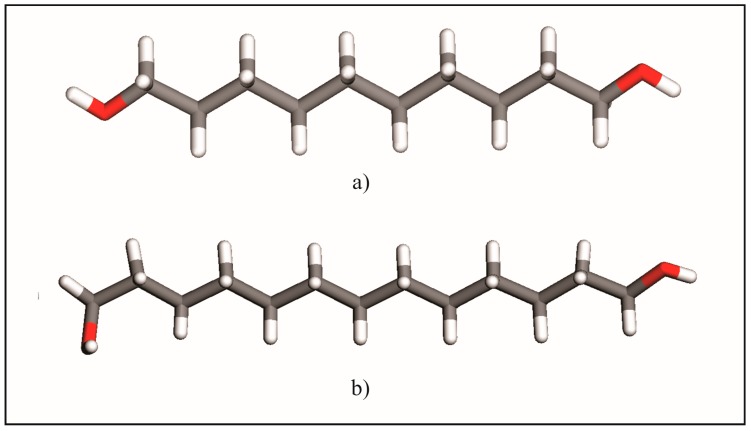
Conformers in long-chain 1,ω-alkanediols series: (**a**) TTt-TTt and (**b**) TTt-TGt.

**Figure 3:**
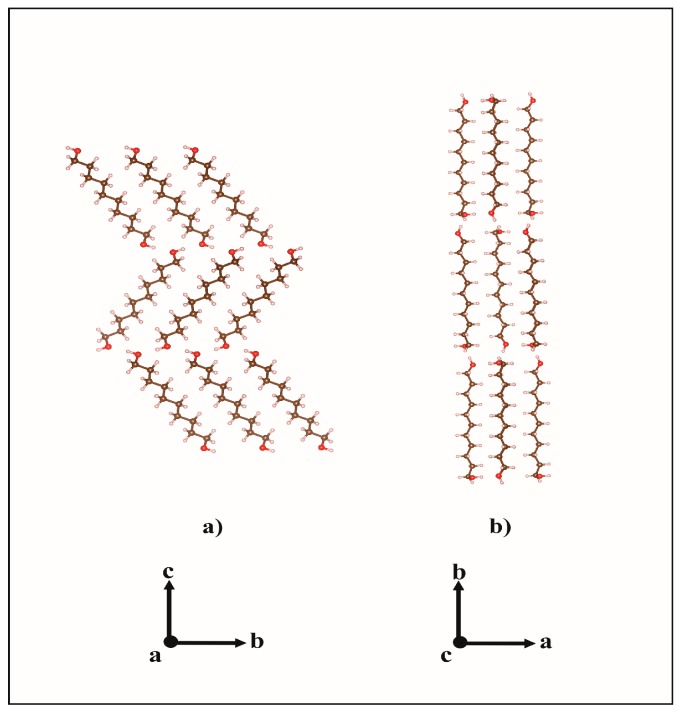
Molecular packings of (**a**) DODOL structure along *a* and (**b**) UNDOL structure along *c*.

These results corroborate the earlier description [3,5] of the odd-numbered member conformers whereby its molecules show two kinds of terminal conformations, a *trans* conformation (Figure 2a) relative to the all-*trans* carbons skeleton (TTt) and, in the opposite ending, a *gauche* conformation (TGt), as is illustrated in Figure 2b. Molecules in the crystal structures of DEDOL and DODOL are arranged in a *herringbone* motif (Figure 3a), with interlayer hydrogen bonds disposed as a *zigzag* infinite chain, in a two-dimensional network on plane(10-4). In odd-numbered 1,ω-alkanediols, the *trans-gauche-trans* sequences form interlayer and intralayer hydrogen bonds disposed alternatively along an infinite 4-members chain. This latter three-dimensional network of hydrogen bonds may be irreducibly described by a basis set of the principal directions [001], [010] and [100], where [100] is the direction of the infinite hydrogen-bond chain. The conformer TTt-TGt as well as the pattern of hydrogen bonds chain result, in this case, in an apparent parallel arrangement of molecular *backbones* (Figure 3b).

**Figure 4:**
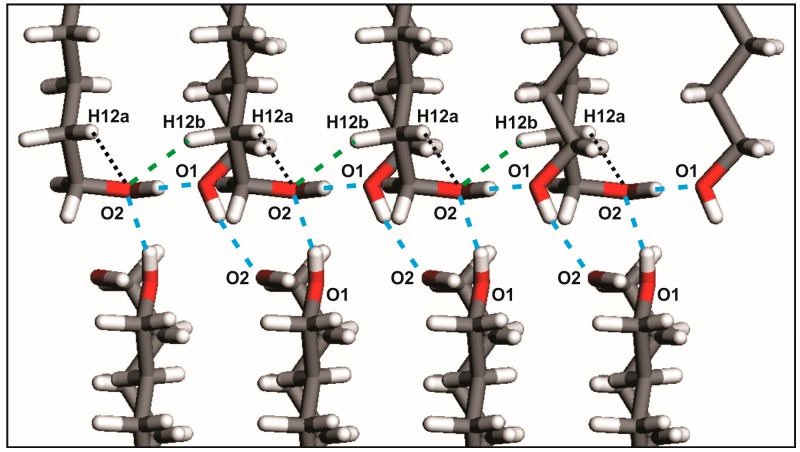
Principal non-bonded interactions in UNDOL and TRDOL. Green discontinuous line: O-H···O hydrogen bond interactions; blue discontinuous line: C-H···O hydrogen bond interaction. Dotted black line: intramolecular C-H···O interaction.

Each hydrogen bond in both patterns links two molecules symmetrically related by a screw axis 2_1_. On the other hand, weak hydrogen bonds C-H···O in odd-numbered members UNDOL and TRDOL are encountered between the C2-H bond of the -TGt isomer of a molecule and the hydroxyl Oxygen -O1- of the same -TGt segment of the contiguous molecule along direction *c* (Figure 4). This behavior suggests that this weak interaction aims to stabilize both the *gauche* defect and, subsequently, the OH···O intermolecular pattern. Namely, it should be responsible for redirecting the OH···O into the intralayer interaction, leaving only one interlayer hydrogen bond. From an intramolecular point of view, the -TGt isomer is also stabilized by a C-H···O interaction between hydroxyl oxygen -O1- and a methylene hydrogen of the β-carbon (C2) in the alkyl chain (Figure 4). The corresponding van der Waals distances are 2.60 and 2.61 Å, for UNDOL and TRDOL, respectively, which are very close to the sum of van der Waals radii of oxygen and hydrogen atoms [24]. So far, the oxygen atom in the -TGt molecular ending shows a multifunctional crystal packing behavior (Figure 3): it simultaneously acts as donor and acceptor in both hydrogen bonds O-H···O, as well as being involved in the stabilization of the *gauche* defect via the intralayer hydrogen bond interaction C-H···O, and the intramolecular interaction O···H (C_β_). The chain parity effect influences molecular conformation by the stabilization of conformer TTt-TGt in odd-numbered members; that is to say, the combination of isomers with more stability [24] and the trans-gauche-trans sequence is the more effective way to maintain the chain pattern of O-H···O. The structures with even-numbered alkyl chains guaranties the stabilization of a chain pattern of hydrogen bonds by an all-*trans* conformer or TTt-TTt, i.e., *trans*-*trans* sequence, without C-H···O hydrogen bonds or determinant conformer-stabilizing van der Waals interactions.

The results of the hydrogen bond geometrical and energy analysis are shown in Table 1. The energy associated to interaction C-H···O fall within the range of weak hydrogen bonds [25]: −2.19 and −3.46 kJ/mol, for UNDOL and TRDOL, respectively. The mean of total hydrogen-bond energy per mol of molecules for the alkanediols series results in a value of E_hb_ = −28.5 ± 0.8 kJ/mol. The error bar is selected by parallelism with the energetic range accepted for strong hydrogen bonds [25]. The value of E_hb_ determined here is presumed to be characteristic for the 1,ω-alkanediols series and represents a constant energetic component in the general expression of the carbon-number-dependent lattice energy, i.e., E_latt_ = f (*n*_c_).

The packing index appears as an effective parity-dependent coefficient, in agreement with the fact that even-numbered members clearly show a denser crystal packing: 71.9 vs. 66.2% (average values), for even and odd-numbered members, respectively. This denser packing or high effectiveness on molecular inter-grooving in even-numbered members is also depicted by the average shortest distances in the C···C interaction of parallel molecules: 4.54 Å for DEDOL and 4.20 Å for DODOL, in agreement with the report of Thalladi et al., for 1,ω-alkanediols of a shorter chain length [1]. The mean value for the UNDOL was 5.14 Å and 5.12 Å for TRDOL.

These two ranges of packing indexes and subsequent C···C interaction distances are related to the values of *herringbone* angles of both polymorphs. *Herringbone* angles (angles between two longitudinal planes of adjoining molecules of a different layer measured along the *a* axis) are 105.81° for DEDOL and 103.02° for DODOL. In odd-numbered 1,ω-alkanediols, there is a mutual and slightly opposite rotation of antiparallel molecules about direction *c* that reduces the theoretical angle of 180° in a parallel array, leaving angles of 170.21° and 175.77° for the UNDOL and TRDOL, respectively. Thus, *herringbone* angle is also an important feature for the geometrical description of structures of odd-numbered members; they also present an angle different from 180°. So far, the herringbone “level” of the molecular array is intrinsically related with hydrophobic intermolecular interactions, and it is presumed that the control parameter consists of maintaining a constant value of hydrogen-bond energy per isomer. Thus, the hydrogen-bond patterns play an important role to control the degree of molecular packing and the molecular arrangement of the *herringbone* motif. It is to say that a *herringbone* angle close to the parallel array (i.e., 180°) implies longer interatomic C···C distances and a less effective molecular packing, in order to maintain the hydrogen-bond energy per isomer. This is the case, in fact, of the odd-numbered alkanediols, with the isomer TTt-TGt, and the typical 4-member chain pattern of O-H···O bonds in group *P*2_1_2_1_2_1_. Instead, a 2-member chain strong hydrogen bond pattern in even-numbered members is maintained yielding a more effective crystal packing with a much smaller *herringbone* angle in a monoclinic structure *P*2_1_/*c*.

Lattice energy studies were performed by means of a single-point energy calculation procedure in order to validate the obtained GIXD crystal structures. This validation process consists of comparing the calculated energetic value against the corresponding sublimation enthalpies—Δ*H*_sub_ (298 K) = Δ*H*_tr+fus_ (298 K) + Δ*H*_vap_ (298 K)—, using experimental thermodynamic properties reported earlier—[26], heat capacities of solid phases, and [27] for vaporization enthalpies and temperatures, as well as the fusion properties from our own DSC’s experiments. DSC thermograms of DEDOL, UNDOL, DODOL and TRDOL are shown in four figures within the Supplementary Materials file. The obtained values of calculated lattice energies correspond to the summation between the energetic terms of van der Waals, electrostatic energy and hydrogen bond energy, E_Lattice_ = E_vdW_ + E_Elect_ + E_bb_ − 2*RT*. Values of E_latt_ and related energy components are divided by 4 and 2 for orthorhombic (P2_1_2_1_2_1_, *Z* = 4, for odd-numbered members) and monoclinic (P2_1_/c, *Z* = 2, for even-numbered members) phases, respectively, in order to correlate energetic values within the same series of 1,ω-alkanediols. The comparison can be observed in Table 2, which shows a slight discrepancy between the experimental and calculated results that can be attributed, in general, to the experimental uncertainty [28] and to the approximation with the 2*RT* term. Another reasonable validation is the comparison of crystal structures obtained by GIXD data with that obtained with single crystal X-ray diffraction by means of measure of similitude, Δ [29]. The values of Δ are 0.025, 0.014, 0.079 and 0.013 Å for the 1,ω-alkanediols with *n* = 10, 11, 12 and 13 respectively, obtained with the program COMPSTRU from Bilbao Crystallographic Server [30,31,32]. These values indicate the excellent (good, concerning DODOL) agreement between the obtained GIXD results and those obtained by single crystal diffraction.

The differences between sublimation enthalpy and calculated lattice energy, as well as those related to the measure of Δ for the crystal structure of DODOL can be attributed to a modification of the *herringbone* form. In this case, we found a new polytype of DODOL that shows a value of monoclinic *β* angle only some decimals above 90°. Although the concept of polytypism is commonly encountered in the description of structures of inorganic [33] and organometallic [34] compounds, its use in the field of the organic crystals is very limited [35]. Polytypism is defined as a special case of polymorphism where structural differences are explained, in general, by a different superposition of layers along one direction. Accordingly, conformational differences between molecules of different layers, as well as different supramolecular motif or synthon are used to identify polymorphs, not polytypes. On the other hand, subtle modifications such as a different stacking sequence of identical molecular layers as well as relative shifts of layers (different superposition) along one or two directions normal to the stacking vector [36] serve to identify polytypes. DODOL shows an identical structure of molecular bilayer than the DODOL determined by single-crystal X-ray diffraction [4], but a slight difference on *herringbone* angle arises concomitant with that of lattice energy. It is important to note here that van der Waals distances on C···C interaction for DODOL are rather different to those from DEDOL. Moreover, the value of the lattice energy of DODOL is not aligned with the other members of the subfamily on the linear relationship E_latt_-*n*_c_, where *n*_c_ is the number of carbon atoms in the hydrocarbon chain. In other words, DODOL is a polytype with energetic and structural differences by comparison with the dominant polytype (i.e., namely polytype I within monoclinic and orthorhombic polymorphs), either for even or odd-numbered members. Its *β* value in the monoclinic cell is an indirect indicative of different polytype, and its value close to 90° leads to consider the structure as *pseudo-orthorombic*. This assumption links well with the first studies [37,38] about organic polytypism where the different polytypes were assigned using the modifications on molecular tilting in aliphatic long-chain compounds, and their subsequent changes in monoclinic angle. Remarkably, this work does not propose the tilt of molecules within a layer for distinguishing polytypes, but the *herringbone* angle that is presented is a key tool for this propose.

### 2.2. Preferred Orientation Analysis

The flat specimen and the fixed holder, as well as the proper reflection geometry of the GIXD experiments, lead us to consider that the registered diffraction intensity is probably affected by textural effects. The preferred orientation r March parameter was refined for a unique Bragg reflection during the first attempts of structural refinement. The resulting fit was substantially improved though some differences between both patterns were still significant over other reflections. In view of that, a second preferred orientation was added (when necessary) and both March parameters were refined together with other variables, namely, unit cell dimensions, asymmetry parameters, global isotropic thermal displacement parameter and zero shift.

**Figure 5:**
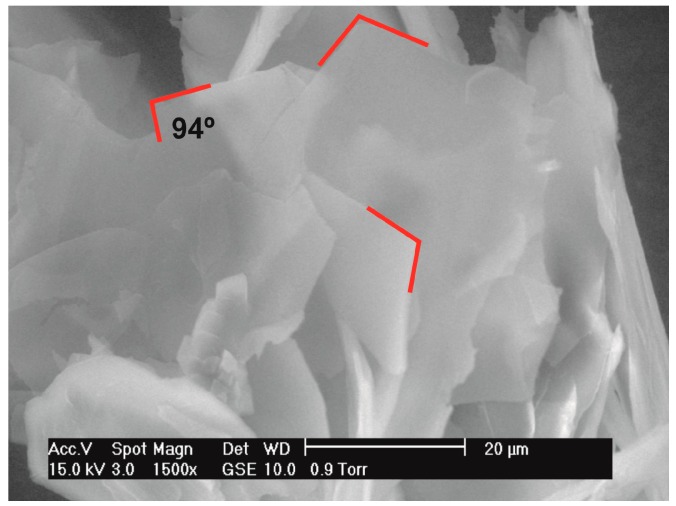
SEM image of angle between oriented planes in DEDOL samples.

**Figure 6:**
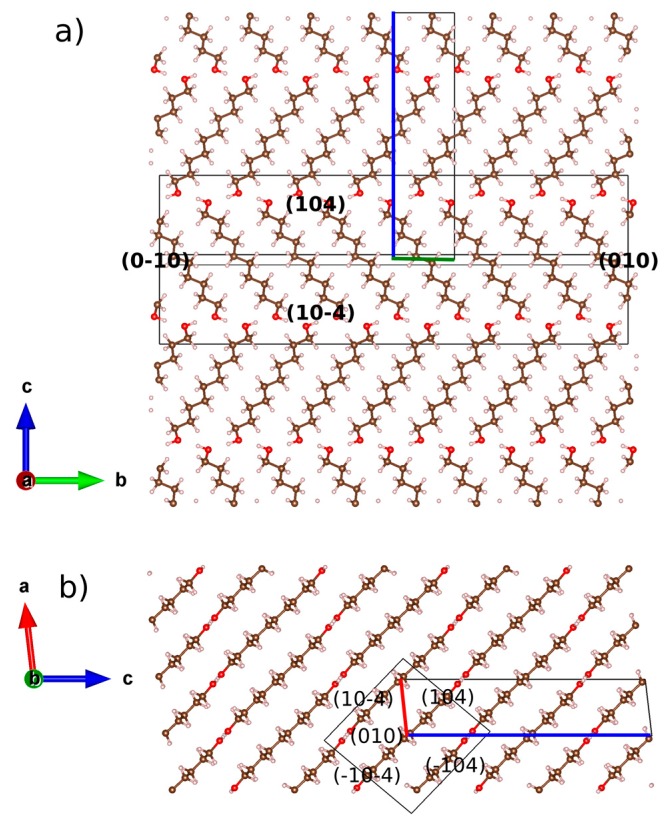
Scheme of the relationship between the crystal structure of DEDOL and the associated combined form {-104}/{104} and pinacoid {010}, along (**a**) *a* and (**b**) *b* axes. Boundary of the unit-cell is drawn with the corresponding colors of the compass. Crystal form is represented by a wireframe with (hkl) of crystal planes. Crystal forms and structure are not at same scale.

The planes (-104) and (208) showed preferred orientation in the DEDOL sample. The latter is one plane among those of the family {104}, which connects methylene groups of adjacent molecules in the molecular layer. Both planes showed a value of r-March parameter <1, indicating a double platelet-like orientation of the crystallites of the sample. The acute angle between both planes is 86.04°, showing a relatively significant difference with respect to the value of the monoclinic angle *β* of the unit cell expressed after a conventional setting: 83.30°. About this comparison, it is important to note that the transformation of unit cell to a setting of parameters expressed in a conventional protocol is carried out since the original parameters are obtained from the whole pattern fitting process using a reduced cell with the obtuse angle *β* closest to 90° (Table 3). The platelet-like nature of the orientation model characterized by two preferred planes lead us to speculate about the occurrence of a likely columnar habit of the crystallites, where the angle between both planes would determine the shape of a third surface. On the assumption of equivalence between the angle between oriented planes (-104) ^ (208) and *β* of the unit-cell, the scanning electron microscopy (SEM) image in Figure 5 corroborates this assumption. A scheme of the combined form {-104}/{104} and pinacoid {010} encountered in DEDOL from the March-Dollase approach is illustrated in Figure 6. The DODOL presented preferred orientations over the planes (104) and (1 0 10). The overlapping of peaks in the 1,ω-alkanediols GIXD patterns was evident, and the selection of the preferred oriented planes in DODOL was not straightforward because of the close presence of the Bragg reflections in crystallographic planes (104) and (-104) with a slight difference of 0.008° in 2θ. Therefore, the correct selection of the strongest preferred oriented plane was done with the help of the isomorphic crystal structure of DEDOL wherein the most intense reflection plane is undoubtedly for the (-104) plane. In fact, the refinement of the March parameter for (-104) in the DODOL pattern improves the fit. The plane (1 0 10) passes through the molecular center and grouped the binary axis of the C_2_ molecules. Both oriented planes in DODOL showed an r-March parameter that also indicates a platelet-like behavior (Table 3). The angle (-104) ^ (1 0 10) results in a value of 77.54°, very close to the value of *β* of the conventional unit-cell, that is 78.45°. Thus, in this case, it seems reasonably to explain the rhomboid surfaces of the crystallites on the basis of the value of *β*. Textured fractions for reflections (-104) and (1 0 10) are 55% and 44%, respectively, indicating a similar preponderance of both platelet-like orientation models.

Our results show that even-numbered members crystallize in a platelet-like fashion with the molecular layers disposed parallel to the plate plane (-104). Otherwise, the platelet-like behavior is also represented by planes (208) –face {104}– and (1 0 10) for DEDOL and DODOL, respectively. This latter statement is in agreement with the extended idea that long-chain layered organic compounds present a packing model where mono or bimolecular layers stack perpendicular to the platelet surface e.g., [39,40]. The planes where the two alternated orientations of the molecular stacking or herringbone motifs can be projected explicitly in face {010}, or normal to bisector of the herringbone angle (see Figure 3a) did not present preferred orientation effects. This latter assumption leads us to infer the habit of the crystallites in a columnar shape, where the more developed planes are those forming the open prisms and the smallest crystal surfaces correspond to the pinacoid. A common irreducible axis zone from the intersection between both prismatic forms (i.e., {-104}/{104} and {-104}/{1 0 10} for DEDOL and DODOL, respectively) was encountered: [010], that is coincident with the parallel direction of the molecular layer and, in view of the crystalline habit, the predominant growing direction (Figure 6). These planes, (010), are rhomboids with the acute angle close to the acute *β* angle of a conventional monoclinic cell.

Note that for the first refinement cycles of preferred orientation in UNDOL, the (200) and (0 12 1) planes appear as possible preferred orientations. A third preferred oriented plane, (101), was then added according to the unique oriented plane present in TRDOL and refinement is considerably improved. Face {101} contains the carbon atoms in the skeleton of the hydrocarbon chain and the respective r-March value >1 indicates the needle-like orientation effect. When only this plane is considered in the preferred orientation analysis as, for example, in the case of TRDOL, the uniaxial orientation is represented by the stacking of bilayered molecular motifs along a direction normal to the surface of the sample. The needle axis is on the longitudinal direction of molecular skeleton. The needle-like orientation associated to (101) plane has not been encountered in the other two oriented planes in UNDOL, (200) and (0 12 1), that showed a r-March parameter of 0.9041 and 0.4675, respectively and, consequently, a platelet-like preferred orientation. The former value, with 0.9 < r < 1, allow one to assume that the (200) plane was slightly oriented because the March parameter was close to 1, i.e., a quasi-randomly oriented sample as a function of the considered plane. Also, (0 12 1) is the plane for methylene groups and, from a preferred orientation point of view, is similar to plane (208) of DEDOL, since it is nearly perpendicular to the superposition of bimolecular layers. In view of its textured fraction, 46%, this plane is the predominant platelet-like orientation pattern.

**Figure 7:**
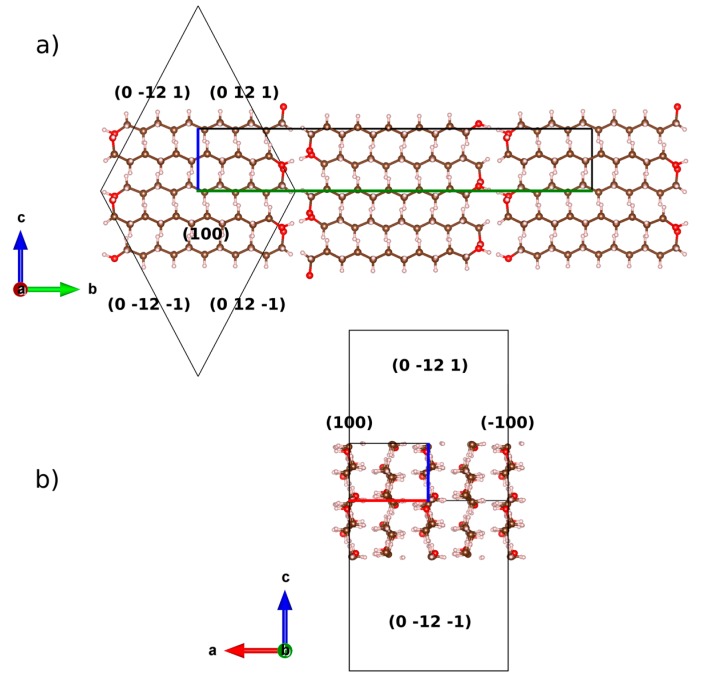
Scheme of the relationship between the crystal structure of UNDOL and the associated combined form {100}/{0 12 1}, along (**a**) *a* and (**b**) *b* axes. Boundary of the unit-cell is drawn with the corresponding colors of the compass. Crystal form is represented by a wireframe with (hkl) of crystal planes. Crystal forms and structure are not at same scale.

**Figure 8:**
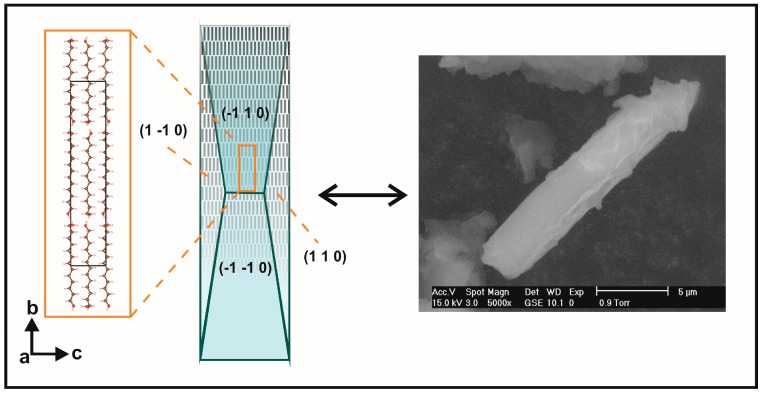
Comparison between deduced crystal morphology {110}/{101} and scanning electron microscopy ( SEM) microphotograph of TRDOL. Arrangement of molecules is illustrated by black bars inside the wireframe of crystal form. Crystal forms and structure with unit-cell are not at same scale.

Planes (101) for UNDOL and TRDOL show a value of March parameter >1, indicating a needle-like model of orientation. A uniaxial model of needles is in agreement with the preferred orientation model of TRDOL. The main difference between UNDOL and TRDOL arises from the fact that UNDOL presents a multiaxial (3) pattern of preferred orientation, with a mixture of needle and platelet-like, i.e., rhombic prism {0 12 1} and pinacoid {1 0 0} models (Figure 7), whereas TRDOL shows a pure uniaxial needle pattern (Figure 8). A great maximization of SEM image (Figure 8) shows the needle habit comprising a combination of forms: {1 1 0} with {1 0 1} or {1 0 0}. This relative disagreement on morphology of structures of the same polymorph may be due to a solvent effect in the recrystallization process.

To summarize, March parameter results (Table 3) on preferred orientation analyses show that the crystal shape of even-membered 1,ω-alkanediols are platelet-shaped while the odd numbered 1,ω-alkanediols are predominantly needle-shaped. This behavior demonstrates a relationship between the molecular arrangement and the crystal shape. The mutual textural behavior in each polymorphic case appears as a facile and indirect way to demonstrate that related structures are isomorphic. Slight differences on preferred orientation models within the same crystal form are possible and their identification is related with different monoclinic angles or crystal growth conditions (i.e., solvent effect).

## 3. Experimental Section

### 3.1. Samples

Samples of 1,10-decanediol, DEDOL (98% pure) and 1,12-dodecanediol, DODOL (99% pure) from Sigma-Aldrich were used as received. Then, 1,11-undecanediol (UNDOL) and 1,13-tridecanediol (TRDOL) were synthesized from the corresponding dicarboxylic acids by a reduction procedure reported elsewhere [41]. First, *N*-methylmorpholine (1.01 mL, 9.24 mmol) and ethyl chloroformate (0.88 mL, 9.24 mmol) [42] were mixed with a dissolution (2 °C) prepared with 1 g of undecanedioic acid (4.62 mmol) in 50 mL of THF. This mixture was stirred for 15 min and a precipitate of *N*-methylmorpholine hydrochloride was obtained. This precipitate is filtered, washed with THF and mixed at 2° C with 7 mL of an aqueous sodium borohydride solution (0.56 g, 14.78 mmol). Then, 120 mL of water was added after degassing the solution. Finally, ethyl acetate was used to obtain the organic extract that, subsequently, was dried over sodium sulfate. Purified UNDOL is obtained for concentrating them under reduced pressure and recrystallized in ethyl ether. All this synthesis was also used to obtain TRDOL but, in this case, 1,13-tridecanedioic acid was selected as the starting product. TRDOL was recrystallized in toluene instead of ethyl ether in order to evaluate possible changes on crystallite morphology of isostructural compounds.

### 3.2. GIXD Data Collection

GIXD experiments were performed using a RIGAKU Ultima-IV diffractometer (40 kV, 30 mA) with Cu Kα_1,2_ (λ_1_ = 1.5406 Å, λ_2_ = 1.5443 Å) radiation and asymmetric configuration (fixed incident angle at 5°). A primary graphite monochromator was used. Then, 2θ intensities were collected with a scintillation detector. A parallel beam was selected with a multilayer mirror. Collimation of the incidence beam was achieved by two divergence slits, both of 1.0 mm. In order to avoid eccentric error, a type of Söller slit (0.5°) that acts as a parallel slit analyzer was positioned between anti-scatter and receiving slits in the secondary diffracted beam. The sample was dusted carefully and then slightly pressured in a circular way with a sheet of glass. The holder was made of glass. Then, 40 min experiments were performed as follows: 2–70° 2θ range, step of 0.02° 2θ and ~0.7 s per step. The condition of infinite thickness for a flat specimen is accomplished in view of the calculated value of *t*, a maximum X-ray penetration depth of ~0.8 mm [43], smaller than the 2 mm-depth of the glass holder. Thus, it is not expected to find any scattered intensity from glass substrate. Intensity correction due to asymmetrical configuration of the experiment [44] was considered on Rietveld and profile refinements [45]. Measurements were performed at room temperature without considering the heating caused by X-rays.

### 3.3. Thermal Analysis

In order to establish fusion temperatures and enthalpies, several thermograms were obtained from DSC (Differential Scanning Calorimetry) experiments carried out with a METTLER Toledo DSC822a calorimeter. Powdered alkanediol samples were tested four times with a weight of 4.0 ± 0.1 mg and introduced in hermetically sealed high purity aluminum crucibles with a capacity of 40 μL. Measurements were done in a N_2_ atmosphere with a constant flow of 40 mL/min and a rate of 2 K/min on heating and cooling.

## 4. Structure Determination

### 4.1. Indexation and Determination of Space Groups (i), and Whole-Pattern Fitting (ii)

Program DICVOL04 [46] was used to index the polycrystalline GIXD patterns. Also, 20 reflections with angles smaller than 60° yield the unit cells with monoclinic and orthorhombic symmetry for both even and odd number of carbon atom members, respectively. The more reliable figures of merit [47,48] and the associated unit cell dimensions are shown in Table 4. The systematic absences were compatible with space group P*2*_1_/*c* for DEDOL and DODOL. In the case of UNDOL and TRDOL, the well-matched space group was P*2*_1_*2*_1_*2*_1_, in accordance with single-crystal structures reported earlier [3,5].

The refinement of profiles was carried out by a Profile Matching [49] option (whole-pattern fitting), implemented into the FULLPROF program [50]. A Pseudo-Voigt function [51] is selected to analyze peak profiles, setting up an asymmetry correction to angles lower than 25° in 2θ by means of the Berar and Baldinozzi method [52]. Unit cell, FWHM and shape parameters were refined. Background was determined by linear interpolation of manually selected points by means of the program WinPlotr [53]. The Profile Matching procedure resulted in good values for the R factors (R_wp_ and R_p_ ≤ 4.0%). The refined unit cell parameters were used in the subsequent calculation of energy minimization.

### 4.2. Isomorphic Molecular Replacement (iii), and Lattice Energy Minimization (iv)

In order to assure a successful lattice energy minimization, the structural model may be built from unit cell parameters and molecular geometries (asymmetric units) that result in an energetic value close to the corresponding experimental local energetic minimum. Unit cell parameters were obtained from the Pattern Matching procedure. Two possible molecular geometries are represented by the *gauche* and *trans* configurations of hydroxyl groups about the all-*trans* hydrocarbon chain (Figure 2). Their occurrence depends upon chain length and recrystallization rate [13], as demonstrated in the previous reports on these structures [1,2,3,4,5,6,7,8,9,10,11,12,13].

Although the four crystal structures have been reported earlier, this work tries to show the effectiveness of the molecular replacement procedure using crystal structures of compounds with higher chain lengths as reference data. The selected structures may present the same space groups and conformations to those found in the studied compounds. An adequate model for DEDOL and DODOL was the 1,18-octadecanediol [10], while for the case of UNDOL and TRDOL, the adopted model was the 1,17-heptadecanediol [9]. Note that *P2*_1_/*c* was used instead of *P2*_1_/*a* for both DEDOL and DODOL structures for comparative purposes. For these even-numbered members, the molecular model within the asymmetric unit is a half molecule with symmetry *C*_2_. The defect *gauche* on one end of molecules in odd-numbered members (UNDOL and TRDOL) of 1,ω-alkanediols implies the lack of intramolecular symmetry (*Z*’ = 1). The new models of crystalline structures are obtained using the mol_TPCR console utility in the Fullprof program [47]. The atom coordinates are derived from the distances, bond and dihedral angles of a Z-matrix of the crystal in accordance with the crystal structure of reference and maintaining the same fractional position (i.e., Xc, Yc and Zc) on atom O1, as well as the orientation (i.e., Euler angles, β and γ) of the asymmetric unit. For instance, Hydroxyl groups (-OH) were adjusted with geometric fundamentals such as torsion angles (HO-C1-C2-C3) of ~180° for the conformation *trans* and ~−60° for the *gauche* conformation, giving bond distances O-C of 1.42 Å. In the resting methylene groups, the distances and angles generated through the original all-*trans* models were fixed.

Lattice energy minimizations were applied on the structure models using the DREIDING model (generic force field for molecular simulations) [54] force field that is implemented in the program for energy minimization and geometric optimization named GULP (general utility lattice program) [55]. In this approach, interactions between molecules are modeled by a combination between van der Waals and electrostatic (Coulomb) energy terms. An additional energetic component associated with a hydrogen bond was added to the original energetic terms. The convergence criterion was set to 10^−3^ Kcal/mol Å and the Gasteiger method was applied to the calculation of charges [56].

### 4.3. Rigid-Body Rietveld Refinement (v)

Once the molecular replacement and the subsequent energetic minimization of all the crystals are performed, the starting structural models are used to start the Rietveld refinements [57,58] in the FULLPROF program [50]. Refinements were carried out in an angular range of 2.0–70.0° in 2θ with slight constraints in angles and bonds. The hydrocarbon chain was treated as a quasi-rigid body (including all the hydrogen atoms) allowing the free rotation of the terminal groups C-O-H. The center of the rigid-body is expressed in orthonormal coordinates (x_c_, y_c_ and z_c_). This absolute position and the Euler angles describing the molecular body orientation (θ, ω, ψ) were refined. In the molecule of odd-numbered members, the central C atom acts as the center, whereas the midpoint of the distance separating two central C atoms in the hydrocarbon chain of DEDOL and DODOL is the corresponding center point. During the initial cycles of Rietveld refinement, the profile parameters and unit cell were kept constant, allowing refinement of the scale factor, zero shift and the intensity of the background points.

At the end of refinement, the correction of preferred orientations was applied using the March-Dollase function for selected planes [19]. The final refinement of all parameters (scale factor, global isotropic displacement, thermal parameter and preferred orientations) gives little difference between the experimental and calculated diffraction patterns, as the goodness-of-fit and R indicators show (R_wp_, R_p_ and χ^2^) [59]. In Table 3, the results of the Rietveld refinement are summarized. Cif’s of the refined structures can be obtained as supplementary material in the corresponding link.

## 5. Conclusions

Crystal structures of 1,ω-alkanediols with 10, 11, 12 and 13 carbon atoms in the skeleton chain were determined from GIXD data in combination with lattice energy minimization by means of a force field that includes the hydrogen bond component. The starting molecular model was obtained from larger compounds in the series. Rietveld refinement was done by a rigid-body approach. The study of preferred orientation in the final stage of refinement revealed a different morphological pattern as a function of the crystallographic form. That is, even-numbered members occur in a platelet-like habit, whereas UNDOL and TRDOL are needle-like. This preferred orientation analysis guarantees success in the structural determination using GIXD data, and is a valid approach to apply to the microstructural characterization of thin films on the air-water interface for this kind of layered amphiphilic series. In general, this work contributes to the elucidation of bulk crystal structures of amphiphilic layered organic compounds. Cooperation between a pattern of strong hydrogen bond and weak ones stabilizes the isomer *gauche* at one end of the odd-numbered alkanediols. Otherwise, a second pattern of a strong hydrogen bond compatible with the all-*trans* conformer (even-numbered members) is more effective on molecular packing. The present results are a complement of the single crystal X-ray data reported elsewhere, and constitutes the first report about long-chain 1,ω-alkanediols structures obtained from powder samples. In this sense, the relevance of this work arises from the fact that its structural refinement procedure is facile and adaptable to low-resolution diffraction experiments.

Differences in the energetic values of lattice energies and in the structural comparative index allow one to identify a new *pseudo-orthorhombic* polytype in the DODOL compound. The comparative study shows significant structural differences within a small energetic range, suggesting that in future work, the morphological features could be analyzed with respect to the method used for the achievement of crystalline powders of 1,ω-alkanediols (e.g., different solutions).

## Figures and Tables

**Table 1:** Geometry of hydrogen bonds in the 1,ω-alkanediols crystals. Atoms labels are preserved such as they appear in Supplementary Materials.

Crystals	D-H···A	D-H (Å)	H ···A (Å)	D···A (Å)	D-H···A (°)	E_hb_ (kJ/mol)
DEDOL	Oh-Ho···Oh ^i^	0.97	1.85	2.8148	176	−28.15
UNDOL	O2-H2o···O1 ^ii^	0.96	1.75	2.7063	170	−27.15
	O1-H1o···O2 ^iii^	0.97	1.83	2.7968	174	−28.19
	C2-H2a ···O1 ^iv^	0.95	2.68	3.4764	142	−3.19
DODOL	Oh-Ho···Oh ^i^	0.96	1.86	2.8254	177	−28.14
TRDOL	O2-H2o···O1 ^ii^	1.08 ^a^	1.67	2.7440	171	−25.37
	O1-H1o···O2 ^iii^	0.82 ^a^	1.94	2.7470	165	−27.82
	C12-H12b ···O2 ^iv^	1.09	2.54	3.4758	144	−3.46

Symmetry codes: (i) 2 − x, −1/2 + y, ½ − z; (ii) 2 − x, ½ + y, −½ − z; (iii) ½ − x, −y, ½ + z; (iv) x, y, −1 + z. ^a^ Values notably deviated from DFT mean calculated values for O-H bonds (1.001 ± 0.001 Å) [21,22].

**Table 2:** Calculated lattice energies and experimental sublimation enthalpies of the 1, ω-alkanediols.

	ΔH_tr+fus_ (298 K) (kJ mol^−1^) ^a^	T_fus_ (K)	ΔH_vap_(298 K) (kJ mol^−1^)	ΔH_sub_ (298 K) (kJ mol^−1^)	E_latt_ at (298 K) (kJ mol^−1^)
DEDOL	37.17 ± 1.58	343.27 ± 0.60	120.2 ± 4.9 [27]	157.37 ± 5.15	−164.53
UNDOL	36.02 ± 1.42	338.85 ± 0.11	131.7 ± 4.1 [27]	167.72 ± 4.34	−167.07
DODOL	47.45 ± 1.48	352.52 ± 0.35	130.5 ± 5.7 [27]	177.95 ± 5.80	−165.78
TRDOL	41.60 ± 1.37	351.27 ± 0.43	132.8 ± 7.8 [27]	174.40 ± 7.90	−170.68

^a^ ΔH_tr+fus_ is the total enthalpy of fusion, including transition, whenever pertinent, and fusion, on heating experiments (see Supplementary Materials).

**Table 3:** Crystal data from the Rietveld structural refinement.

Crystal Properties	DEDOL	UNDOL	DODOL	TRDOL
Empirical formula	C_10_H_22_O_2_	C_11_H_24_O_2_	C_12_H_26_O_2_	C_13_H_28_O_2_
Formula weight	174.28	188.31	202.34	216.36
Range of 2θ (°)	2–70	2–70	2–70	2–70
Crystal system	Monoclinic	Orthorhombic	Monoclinic	Orthorhombic
Space group	P*2*_1_/*c*	P*2*_1_*2*_1_*2*_1_	P*2*_1_/*c*	P*2*_1_*2*_1_*2*_1_
*a* (Å)	4.9570 (7)	7.1154 (8)	4.9665 (8)	7.1847 (9)
*b* (Å)	5.1820 (9)	32.4798 (3)	5.1895 (9)	37.6468 (4)
*c* (Å)	21.2361 (3)	5.1402 (6)	24.5088 (4)	5.1239 (7)
*β* (°)	96.6938 (4)	90.000	90.7523 (5)	90.000
Volume (Å^3^)	541.776 (9)	1187.93 (5)	630.547 (8)	1385.91 (7)
Z	*2*	*4*	*2*	*4*
U ^a^	0.21474 (5)	0.06757 (3)	0.08719 (2)	0.4460 (1)
V ^a^	−0.04840 (9)	−0.02228 (2)	−0.0435 (1)	−0.02944 (5)
W ^a^	0.10096 (3)	0.1423 (1)	0.09275 (3)	0.10261 (2)
X ^b^	0.00316 (5)	0.00404 (2)	0.00535 (3)	0.00809 (4)
Eta0 (*µ*_0_) ^b^	0.6954 (2)	0.6982 (6)	0.6897 (6)	0.6925 (4)
R_p_ (%)	6.70	6.88	6.98	7.51
R_wp_ (%)	9.30	8.25	9.22	8.75
χ^2^	4.82	4.80	6.47	7.46
*U*_overall_ (Å^2^)	0.1260 (3)	0.4674 (2)	0.0556 (2)	0.1340 (3)
*r March (hkl)*	0.6068 (-104) 0.4434 (208)	1.1816 (101) 0.9041 (200) 0.4675 (0 12 1)	0.6582 (-104) 0.6321 (1 0 10)	1.3323 (101)
Chemical formula	C_10_H_22_O_2_	C_11_H_24_O_2_	C_12_H_26_O_2_	C_13_H_28_O_2_
Formula weight	174.28	188.31	202.34	216.36

^a^ FWHM = $\sqrt{(W+V\tan\theta +U\tan^{2}\theta }$ Caglioti´s law for the width of the peak. ^b^ Eta = Eta0 + X^2^θ, for a pseudoVoigt profile.

**Table 4:** Cell parameters extracted from indexed diffraction patterns of GIXD for the studied 1,ω-alkanediols.

	*A* (Å)	*B* (Å)	*C* (Å)	*Α* (°)	*Β* (°)	*Γ* (°)	F (20) ^a^	M (20) ^a^
DEDOL	4.9355 (3)	4.6817 (8)	20.4366 (3)	90	95.617 (6)	90	24.0	24.5
UNDOL	6.9843 (8)	32.1392 (6)	4.9418 (7)	90	90.000 (2)	90	17.3	17.8
DODOL	5.0704 (2)	5.1411 (2)	23.9209 (5)	90	90.315 (8)	90	15.0	16.5
TRDOL	6.9177 (7)	37.5049 (8)	5.2351 (2)	90	90.000 (4)	90	21.5	18.7

^a^ F_20_ = NNB×1|∆2θ¯| [47] and M_20_ = 1NB×Q202|∆Q¯| [48].

## Supplementary Materials

Coordinates of atoms and DSC’s thermograms of the four compounds (DEDOL, UNDOL, DODOL and TRDOL) are available online.
