# Supplementary material for: From Intermolecular Interactions to Texture in Polycrystalline Surfaces of 1,ω-Alkanediols (ω = 10–13)
Source: Molecules. 2017 Jun 8;22(6):956. doi: 10.3390/molecules22060956 (PMC6152643; doi:10.3390/molecules22060956)
Supplement: Supplementary file 1 [file molecules-22-00956-s001.pdf]

Supplementary Materials: From intermolecular interactions to texture in polycrystalline surfaces of 1,ω-alkanediols (ω = 10 – 13)

G. Luís-Raya, M. Ramírez-Cardona *, G. Luna-Bárcenas *, M. A. Hernández-Landaverde, A. Jiménez-Nieto, I.C. Sanchez

Crystallographic Data for Compounds 1,10-decanediol, 1,11-undecanediol, 1,12-dodecanediol and 1,13-tridecanediol.

Table S1. Atomic coordinates for 1,10-decanediol.

	x	y	z
C5	0.69861	-0.10471	0.18237
C6	0.58970	0.15231	0.15453
C7	0.36596	0.12864	0.09856
C8	0.26343	0.39051	0.07178
C9	0.04993	0.36744	0.01364
H5a	0.77170	-0.22458	0.14478
H5b	0.53408	-0.21330	0.20145
H6a	0.76208	0.26181	0.13953
H6b	0.51304	0.26578	0.19266
H7a	0.19332	0.01887	0.11349
H7b	0.44322	0.01540	0.06040
H8a	0.43724	0.50390	0.05878
H8b	0.17722	0.50058	0.10926
H9a	-0.12503	0.25534	0.02650
H9b	0.13645	0.25610	-0.02357
OH	0.91581	-0.05521	0.23069
HO	0.97767	-0.22368	0.24585

Table S2. Atomic coordinates for 1,11-undecanediol.

	x	y	z
O1	0.97116	0.28322	-0.35224
O2	0.91837	0.71219	0.08107
C1	1.03094	0.28723	-0.09076
C2	0.96868	0.32712	0.03096
C3	1.05120	0.36558	-0.09257
C4	0.97231	0.40513	0.02234
C5	1.04765	0.44427	-0.09737
C6	0.96369	0.48337	0.01717

C7	1.03810	0.52310	-0.09755
C8	0.95220	0.56142	0.02051
C9	1.02576	0.60192	-0.08661
C10	0.93496	0.63917	0.03778
C11	1.00491	0.67987	-0.06224
H1a	1.16105	0.28532	-0.07889
H1b	0.97512	0.26506	0.01158
H1o	0.83552	0.28459	-0.36257
H2a	1.00089	0.32733	0.21003
H2b	0.83422	0.32929	0.01668
H2o	0.96324	0.73824	0.01784
H3a	1.18302	0.36525	-0.06711
H3b	1.02471	0.36513	-0.27386
H4a	1.00177	0.40585	0.20201
H4b	0.84042	0.40512	-0.00023
H5a	1.17960	0.44485	-0.07332
H5b	1.02048	0.44373	-0.27876
H6a	0.99077	0.48381	0.19796
H6b	0.83263	0.48290	-0.00899
H7a	1.16991	0.52412	-0.07200
H7b	1.01177	0.52321	-0.27905
H8a	0.97662	0.56137	0.20242
H8b	0.82055	0.56086	-0.00764
H9a	1.15705	0.60319	-0.05907
H9b	1.00067	0.60278	-0.26873
H10a	0.95897	0.63811	0.22110
H10b	0.80347	0.63791	0.01013
H11a	1.13792	0.68143	-0.04142
H11b	0.97486	0.68253	-0.24104

Table S3. Atomic coordinates for 1,12-dodecanediol.

	x	y	z
C2	0.65156	0.02010	0.16678
C3	0.44365	0.00484	0.12072
C4	0.37696	0.26568	0.09535
C5	0.17089	0.24857	0.04884
C6	0.10233	0.50859	0.02331
H2b	0.57616	0.14953	0.19920
H2a	0.84081	0.10543	0.15088
H3b	0.25558	-0.08248	0.13682
H3a	0.51969	-0.12446	0.08830
H4b	0.56484	0.35460	0.07962
H4a	0.29831	0.39419	0.12767

H5b	-0.01664	0.15886	0.06457
H5a	0.25025	0.12005	0.01661
H6b	0.28977	0.59851	0.00761
H6a	0.02249	0.63709	0.05549
C1	0.72640	-0.23312	0.19132
OH	0.92352	-0.19822	0.23306
H1b	0.54112	-0.31976	0.20903
H1a	0.80462	-0.36657	0.15985
Ho	0.97066	-0.36944	0.24508

Table S4. Atomic coordinates for 1,13-tridecanediol.

	x	y	z
O1	0.41880	0.21729	0.41602
O2	0.45995	-0.22106	0.85303
C1	0.51180	0.18961	0.55346
C2	0.43992	0.15462	0.45336
C3	0.52841	0.12234	0.58341
C4	0.45298	0.08759	0.47693
C5	0.53750	0.05418	0.60162
C6	0.46160	0.02006	0.48761
C7	0.54332	-0.01381	0.60605
C8	0.46854	-0.04756	0.48685
C9	0.55101	-0.08173	0.60260
C10	0.47475	-0.11514	0.47917
C11	0.55051	-0.14939	0.59609
C12	0.46726	-0.18244	0.46910
C13	0.52817	-0.21695	0.59104
H1O	0.45557	0.23721	0.45726
H1a	0.46842	0.19112	0.75279
H1b	0.64130	0.19291	0.52634
H2O	0.31036	-0.21910	0.85786
H2a	0.29103	0.15289	0.47867
H2b	0.45756	0.15646	0.28772
H3a	0.66544	0.12501	0.56687
H3b	0.48721	0.12391	0.78886
H4a	0.31673	0.08585	0.49828
H4b	0.48503	0.08712	0.28760
H5a	0.67653	0.05713	0.58341
H5b	0.51820	0.05469	0.79601
H6a	0.32638	0.01897	0.50127
H6b	0.48259	0.01892	0.30868
H7a	0.69044	-0.01104	0.58079
H7b	0.52388	-0.01370	0.79664

H8a	0.32702	-0.04883	3 0.50188
H8b	0.49922	-0.04855	5 0.29930
H9a	0.69074	-0.08015	5 0.58432
H9b	0.53242	-0.08209	9 0.79202
H10a	0.34008	-0.11661	61 0.49103
H10b	0.50085	-0.11696	96 0.28513
H11a	0.69144	-0.15166	66 0.57620
H11b	0.53911	-0.15049	49 0.76264
H12a	0.33038	-0.18165	65 0.47418
H12b	0.51929	-0.18692	92 0.27182
H13a	0.66210	-0.21691	91 0.60591
H13b	0.47656	-0.23798	98 0.48154
